# Nicotinic Amaurosis—Translation from the French*Having seen the article of M. Guelliot, a short time ago, while looking over the numbers of *Le Progres Medical* for the present year, I have thought that a translation, in full, of his analytical and valuable discussion might be of some interest, as the abuse of tobacco in this country can be scarcely less, I think, than it is among the inhabitants of Paris and Ardennes.—Translator.

**Published:** 1877-12

**Authors:** M. Charles Guelliot


[TRANSLATION.]

### NICOTINIC AMAUROSIS.*

[BY M. CHARLES GUELLIOT.]

Translated from *Le Progres Medical* by A. SIBLEY CAMPBELL, M.D., Augusta, Ga.

It has long been known that those who work in tobacco manufactories are subject to conjunctivitis, and I have had the opportunity of observing this result at the clinic of my learned teacher in ophthalmology, Dr. Galezowski. But less generally known are the disorders of vision produced by the abuse of tobacco-smoking.

In 1847, Wright,† after administering to dogs from thirteen to thirty-two grammes of tobacco mixed in their food, observed that up to the time when a fatal effect was produced they were generally affected with blindness. It is the opinion of Lander, Mackensie and Wordworth,‡ that atrophy of the optic papilla|| is the characteristic lesion of the empoisonment by tobacco. But the majority of authors who have paid attention to this subject have given but little prominence to this lesion of the papilla, and almost all have confounded the nicotinic with the alcoholic amaurosis.

This is easily explained, for tobacco is rarely seen to produce disorders of vision so remarkable as to cause the patient to seek relief at the hands of a physician. Follin,§ indeed, states that he has seen it in two positive cases only. It is this

* Having seen the article of M. Guelliot, a short time ago, while looking over the numbers of *Le Progres Medical* for the present year, I have thought that a translation, in full, of his analytical and valuable discussion might be of some interest, as the abuse of tobacco in this country can be scarcely less, I think, than it is among the inhabitants of Paris and Ardennes.—TRANSLATOR.

† Annals of Hygiene, Vol. 38: Diseases Resulting from the Abuse of Tobacco; by Laycock and Wright. London.

‡ Annals of Hygiene, Vol. 37, second series: On Some Toxic Ambliopiæ; by Dr. Reau.

|| M. Guelliot here refers to the *colliculus seu papilla nervi optici*, described by Kœlliker as "the slight eminence formed by the optic nerve as it enters the eye, and visible on the inner surface of the retina."—TR.

§ *Dictionnaire Encyclopedique.*

rarity of nicotinic amaurosis which has induced me to publish the results of my investigations.

I happened to make my observations in a region where tobacco makes a certain number of victims, and I belive that the comparative frequency of nicotinic amaurosis in Ardennes should be attributed, in the first place, to the great consumption of tobacco there—which is the case, too, in all the departments of the Nord—and perhaps, also, to the Belgian tobacco, which, in a great many villages, has taken the place of the French tobacco. This last supposition agrees with that of Drs. Dickson and Reau,* who state that in Constantinople nicotinic amaurosis is less frequent than in other countries, and they believe that the explanation of this difference should be found in the quality of the tobacco of the East, as it is weaker than the French tobacco.

After having observed at Vouziers several examples of this disease—my attention being first called to it by a judicious and indefatigable observer, Dr. Vincent—I had the good fortune, in Paris, to meet with similar cases which I succeeded in detecting by interrogating all the patients who entered the hospital. I proceed now to report some of these observations, and after these I will give the notes of a case of alcoholic amaurosis, for the purpose of showing what should be, in my opinion, the points of differential diagnosis between the two diseases.

*Observation I.*—D., a glover, about fifty years of age, presented himself at the clinic of Dr. Galezowski for a conjunctivitis contracted some days before. After the consultation, I took him aside and questioned him with a view to the possibility of tobacco intoxication, as I had been in the habit of doing with all the patients whom I had to examine. I then learned that this individual had an affection of which he had not at all complained.

Here are the evidences furnished me: This man smokes from morning till night (forty centimes'† worth of tobacco a

---

* Annals of Hygiene, Vol. 37, second series, loc. cit.

† A *centime* being the hundredth part of a franc or livre, and the franc equal to twenty sous, "forty centimes" would be equal to eight sous, or about eight cents of our currency. The quantity of tobacco implied in the text would, of course, depend very much upon the quality, and upon day). The right eye is completely lost. When this eye began to fail, a year ago, the patient saw everything through a cloud; a little while after everything seemed yellow to him. The other eye was not impaired until afterwards. On this side the central acuteness is destroyed, while the peripheral sensibility is still normal. Besides the central scotoma,* the patient continually sees *muscæ volitantes*, and it also occurs to him, from time to time, to see objects covered over with a yellow tint, but he has never had any pain. Finally, he has never confounded colors, a point on which he gives a perfectly clear account, by reason of the nature of his vocation. In fact, I showed him one piece of ten francs and one piece of fifty centimes;† he distinguished them perfectly. He assures me that he sees less distinctly at night than during the day. I concluded the examination of my patient with the ophthalmoscope: the right papilla is atrophied, the left is certainly anæmic, whiter than in its normal condition. The ophthalmoscope did not reveal anything else.

Finally, on the right side (the side completely lost) the pupil is *contracted* and immovable. This patient, notwithstanding my advice, did not return to the clinic of Dr. Galezowski. I have, therefore, been unable to determine the effect of the treatment, which I prescribed for him—so simple, but, it is true, so difficult to follow—viz: to abstain entirely from smoking.

*Observation II.*—L., aged about sixty-two years, a clerk, entered the hospital Necker, (service of M. Guyon) for an ep-

---

other circumstnnces also; the effect, too, would depend greatly upon the strength, which may vary within the different qualities; but in this country the quantity would perhaps approximate two ounces of fair smoking tobacco. In France the value is no doubt higher than it is here; but enough has been said to infer that the amount consumed was sufficient to produce serious derangement of the nervous system, for we at least know definitely that the patient smoked a pipe "from morning till night."—Tr.

* *Scotoma*, (from SKOTOS) darkness, shadow, obscurity of vision; called also scotasma, scotosis, etc.—Tr.

†The ten franc piece is *gold*, and nearly equal in value to two dollars; the fifty centime, or half franc, piece is *silver*, and nearly equal to ten cents (or, to be more exact, $1.949 and 9.1 cents respectively); consequently, as we should suppose, the two coins approximate each other in size. Hence the propriety of using them as a color test.—Tr.

ithelioma of the rectum. This man was smoking one cigar and from thirty to forty centimes' worth of tobacco every day. After that visit, he informed me that besides the disease which brought him to the hospital, he had also to complain on account of his sight, as it had been considerably impaired for some time.

The *right* eye was attacked two months ago. From the beginning of that period, the patient has seen objects through a mist; he has central scotoma and muscæ volitantes. The mist is becoming thicker and thicker, and from time to time the patient sees objects in yellow (*en jaune*). The left eye was attacked five weeks after the right eye, which is very nearly lost at the present time. On both sides the pupils are *contracted* and immovable. Lastly, the patient *does not confound colors*—he distinguishes perfectly a piece of gold from a piece of silver, and he affirms that he sees no longer *after night arrives*. I will speak further on of the importance which I attach to these last two symptoms. I have not yet been able to make the ophthalmoscopic examination.

*Observation III.*—L., a jailer, smokes thirty centimes' worth of tobacco a day. One day he discovered that he saw everything through a mist. When he endeavored to distinguish anything through the cloud he saw muscæ volitantes pass before his eyes, and the object which he fixed appeared yellow to him. He has never had any confusion of colors, nor has he ever had any headache; this man saw better in the day than at night. At the present time the patient is kept in his bed by a carcinoma of the stomach. By order of his physician, he has abstained from smoking, and his sight is improving from day to day.

*Observation IV.*—M. X., a physician, to whom I made known my investigations, stated to me that for some time he had seen objects through a mist, that he was troubled with muscæ volitantes, that objects often appeared to him *yellow*, that he has never confounded colors, and that as soon as night arrives he sees less distinctly.

*Observation V.*—L., residing at Vaudy, (Ardennes) has been a soldier for two years, then a domestic. During all this time he committed numerous excesses. The right eye was first attacked in 1864; he was at that time a soldier in Africa. The vision of this side is completely destroyed; there is no sensation of light.* The ophthalmoscope shows me, in this eye, a complete atrophy of the papilla and atrophic spots on the choroid. The patient gives me a valuable indication: this eye has been lost in a very short time. The pupil is closely contracted and immovable.

The left eye was attacked one month after the other; the vision on that side is not entirely destroyed; the condition has been stationary for some time.

The ophthalmoscope reveals the same lesions here as in the other eye, but less decided; on this side the pupil is large and movable, and there is no injection of the conjunctiva. The patient has never suffered pain, and he denies altogether syphilitic antecedents. An important fact: he assures me that while he could still see clearly enough to guide himself, his vision was *much less distinct* at night than in the day, and he adds that he has never confounded colors. For a short time since, this individual has not had the means of buying tobacco, and he thinks his sight is a little improved. I advise him to give up his habit entirely—without, however, expecting a great improvement. This is a case of nicotinic amaurosis to an extreme degree.

*Observation VI.*—G., a grain merchant, entered the hospital of Vouziers for a chronic bronchitis. His sight, which, until recently, had been good, became impaired three months ago, both eyes being affected at the same time. There is some diplopia; never, or scarcely ever, any pain; and no lachrymation, unless he is exposed to strong sunlight. This patient *sees better* at night than during the day.

He received, several years ago, a kick from a horse in the left temporal region; the eye was affected for some time, but vision itself was not disordered. Though this patient sees through a mist, he does not complain of muscæ volitantes, but of a simple central scotoma. It has frequently happened to him to confound colors; he sees animals, principally rats, like all the victims of extreme alcoholism. The examination of the inte-

*The term here used in the original ("*phosphenes*") implies specifically the subjective luminous rings, seen in the healthy eye, when the ball is slightly pressed with the finger, appearing on the side opposite the point of pressure.—Tr.

rior of the eye reveals an abnormal whiteness of the two papillæ, perhaps the beginning of atrophy. There is no doubt that I have here a case of alcoholic amaurosis. This man—who smoked, it is true—drank wine all through the day, brandy four times, and absinthe* once a day.

Since that time I have seen the patient again. His sight is but slightly improved, although at the hospital he is deprived of alcohol. I will speak further on of the importance which I attach to this slow progress of the phenomena in the alcoholic amaurosis.

*Conclusions.*—I will state, in the first place, that as Follin† recommends, I have taken care to inform myself as to the quantity of tobacco which each one of my patients smokes in a day. I will state, next, that all the individuals from whom I have taken these observations, smoke a pipe. I have not seen nicotinic amaurosis produced by tobacco in the form of snuff,‡ probably on account of the very slight absorption of the active principle, as has has been remarked in the thesis of Dr. Apostoli.||

If, now, I recapitulate the symptoms which my patients have furnished me, I find:

1. That the nicotinic amaurosis commences always in one

---

* As this drink is not very common in this country, I add the following account of it: "Under this name a *liqueur* is much used in France, consisting of alcohol mixed with volatile oil of wormwood, and some other less active ingredients, especially oil of anise. It has for some time been noticed that the effects of this liqueur differ essentially from those of pure alcoholic drinks, constituting a series of symptoms which have been designated by the name of *absinthism*. * * * From the fatal effects produced by the oil of wormwood, it is highly probable that absinthe is capable of producing immediate fatal convulsions, in quantities in which ordinary spirituous drinks containing a similar amount of alcohol, might be taken with present impunity." (See United States Dispensatory—note to thirteenth edition.)—TR.

†Dictionnaire Encyclopedique.

‡The author does not allude to the effects of tobacco *chewing*, as it is not very fashionable in France, though so common here. It is par excellence the American habit, and in this connection has been most humorously satirized by Charles Dickens, among what he regards as our social obliquities, in his "American Notes."—TR.

||Apostoli—These de Paris, 1872.

eye, and is never bilateral at the first onset, as Hutchinson seems to admit, in the *Lancet* of November 7th, 1863.

2. That the right eye is affected first, although Hutchinson and Apostoli think the contrary.

3. That at the beginning, the patient sees through a mist, which becomes more and more dense.

4. That at the same time with the mist, there exists also a central scotoma—the scotoma being admitted by Apostoli and Wecker,* but not by Follin.

5. That as Wecker says, there is at first a diminution of the central acuteness, while the peripheral sensibility in the beginning remains normal.

6. That I have never met with the pains noticed by Apostoli, nor the cephalalgia of which Follin speaks.

7. That after the appearance of the mist the patients, from time to time, see objects in yellow *(en jaune,)* as Wecker alone has remarked.

8. That there is never any confusion of colors, notwithstanding the views of the majority of authors.

9. That the patients always see less distinctly at night.

10. That the pupils are almost always contracted and immovable.

11. That this disease, when it progresses with sufficient rapidity, may terminate in atrophy of the papilla, as Hutchinson has seen once out of three cases. [*Lancet*, August, 1863.]

In fine, it seems to me that the nicotinic amaurosis and the alcoholic amaurosis may be distinguished from each other, as follows:

1. Because the alcoholic amaurosis begins, primarily, in both eyes, while the nicotinic amaurosis always begins unilaterally.

2. Though the central scotoma exists in both the diseases, the muscæ volitantes are seen in the nicotinic intoxication only.

3. The confusion of colors which is present in the alcoholic amaurosis, *never* shows itself in the nicotinic.

4. Though pain may be present in alcoholic amaurosis, it is not present in that which results from tobacco intoxication.

5. While the victim of alcoholism sees better at night, the

*Wecker—Traite des Maladies des Yeux.

individual afflicted with nicotinic amaurosis sees pretty well during the day, and is no longer able to see when the day has closed.

6. Though both diseases may terminate in atrophy of the papilla, yet the nicotinic amaurosis progresses much more rapidly.

In conclusion, I will state that I have desired to call particular attention to two symptoms of nicotinic amaurosis, which have not been pointed out until now:

1st. The patients see less distinctly at night than during the day.

2d. They never confound colors.

